# Validation and Feasibility of a Consumer-Grade Wearable Sleep Monitoring Device and an Exploratory Evaluation of Sleep Characteristics and Associated Factors Among Nurses: Observational Cohort Study

**DOI:** 10.2196/96912

**Published:** 2026-08-19

**Authors:** Siew Hoon Lim, Fazila Aloweni, Phyllis Ong, Shin Yuh Ang

**Affiliations:** 1 Division of Nursing, Singapore General Hospital, Singhealth Tower Level 15, 10 Hospital Boulevard, Singapore, 168582, Singapore, 65 91802031

**Keywords:** sleep, nurses, shift work, acute care, wearables

## Abstract

**Background:**

Shift work disorder and insufficient sleep are prevalent among nurses, leading to fatigue, reduced well-being, and potential safety concerns. The increasing use of wearable sleep-tracking devices presents an opportunity to evaluate nurses’ sleep quality objectively.

**Objective:**

The primary objective was to evaluate the feasibility of wearable-based sleep monitoring and to obtain preliminary evidence of agreement with validated actigraphy among nurses. The secondary objective was to describe nurses’ sleep characteristics and to examine exploratory associations between sociodemographic characteristics, shift patterns, sleep hygiene, and sleep parameters.

**Methods:**

A 2-phase feasibility observational cohort study was conducted in a tertiary hospital in Singapore. In phase 1, 5 nurses concurrently wore a consumer-grade, wrist-worn Apple Watch Series 10 and a validated actigraph (GENEActiv; ActivInsights Ltd) for 2 weeks. Preliminary agreement between the Apple Watch and GENEActiv was examined using intraclass correlation coefficients. Feasibility was determined through wear-time compliance and data completeness. In phase 2, 50 nurses working rotating or single shifts completed demographic and work-related questionnaires and the Sleep Hygiene Index. Multiple linear regression analyses were performed to examine exploratory associations between selected covariates and sleep parameters, with adjustment for age, sex, BMI, parental status, workplace, total length of service, and sleep hygiene.

**Results:**

The Apple Watch showed preliminary evidence of agreement with GENEActiv for total sleep time, in-bed wake time, and sleep efficiency (intraclass correlation coefficients of 0.95, 0.72, and 0.69, respectively), with high wear compliance and minimal missing data, supporting its feasibility for sleep monitoring. Mean total sleep time was 381 (SD 55) minutes, and mean sleep efficiency was 94.77% (SD 4.11%). Shift nurses reported poorer sleep hygiene than nonshift nurses; however, shift work status was not independently associated with sleep outcomes after adjustment. Higher BMI was associated with shorter total sleep time (unstandardized coefficient *B*=−3.76 min/kg/m²; *P*=.01), reduced rapid eye movement sleep (unstandardized coefficient *B*=−1.18 min; *P*=.03), shorter core sleep (unstandardized coefficient *B*=−2.72 min; *P*=.02), and reduced time in bed (unstandardized coefficient *B*=−4.05 min; *P*<.01=.008). Age was negatively associated with deep sleep duration, with older age associated with less deep sleep (unstandardized coefficient *B*=−1.00 min/y; *P*=.003).

**Conclusions:**

Apple Watch–based monitoring was feasible and showed preliminary agreement with actigraphy. Nurses slept less than recommended, and BMI and age were associated with sleep outcomes in exploratory analyses. These findings support larger studies and workplace strategies to improve sleep opportunity and healthy sleep behaviors.

## Introduction

### Background

Insufficient sleep and sleep disorders are commonly identified health challenges among health care workers who perform shift work [1]. Shift work alters circadian rhythm, causes sleep disturbances, and affects physical and mental health, leading to poor work performance and affecting patient safety [2,3]. This is especially so for nurses who take on night shifts, where the irregularities between their internal circadian rhythms and the work requirements result in alterations in their sleep-wake cycles and light-dark cycles, which lead to a circadian rhythm sleep-wake disorder known as shift work disorder [3]. Shift work disorder is characterized by chronic insomnia whereby individuals experience difficulties in falling asleep and staying asleep, as well as possible excessive sleepiness [4].

Prolonged irregular working hours increase nurses’ vulnerability to the development of sleep disorders, including increased stress and anxiety, depressed mood, and reduced overall mental health [5]. The decrease in sleep quality among nurses has also been reported to increase sick leave days, lower job satisfaction, and burnout [6-8]. Studies have also highlighted possible associations between working night shifts and weight gain, obesity, breast cancer, cardiovascular disease, and metabolic disorders, including type 2 diabetes [9,10]. Regardless of the shift worked, nurses generally report poor sleep quality. Previous studies found that up to 63% of nurses reported poor sleep quality and that 68% highlighted insomnia-related concerns [11].

### Sleep Patterns of Nurses on Shift Work

Nurses largely have no fixed work schedule; they have to manage and adjust their schedules according to their shifts. There is no fixed forward-cyclic rostering schedule. The sleep quality of nurses working on different shifts was evaluated in previous studies [12]. Nurses on the night shift generally had lower total sleep time, higher wake after sleep onset, and lower sleep efficiency during their work days when compared to those who worked day and evening shifts [13]. Nurses with rotational shifts in a counterclockwise rotation of more than 50% of shifts (night to evening to day shifts) reported poor sleep quality as well [12]. Whereas nurses who worked evening rotating shifts reported longer total sleep time when compared to those working day or night rotating shifts, as well as fixed day shifts. Furthermore, nurses on backward rotating–shift schedules also reported higher levels of sleep disturbances and less balanced sleep duration as compared to those on forward rotating–shift schedules [14]. Understanding sleep parameters of nurses working on different shifts is crucial to facilitate the arrangement of work shifts in the best possible manner to lower the impact of shift work on their sleep quality.

### Sleep Hygiene Behaviors

Sleep hygiene primarily involves individual behaviors that facilitate sleep and minimize behaviors that prevent restful sleep [15]. Good sleep habits include avoiding late afternoon naps; minimizing caffeine, alcohol, and tobacco intake; reducing emotionally demanding activities during bedtime; ensuring a quiet and comfortable bedroom environment; and keeping to the same daily sleep schedule [16]. Inadequate sleep hygiene is defined in the International Classification of Sleep Disorders [17] as a type of sleep disorder due to individual activities that interfere with quality sleep and result in reduced daytime alertness.

The review of the literature found few studies exploring nurses’ sleep hygiene behaviors. Recent studies have reported that poor sleep hygiene is associated with poor sleep quality and even with shift work disorder [18,19]. Various common poor sleep hygiene behaviors were identified among nurses, including going to bed at different times, waking at different times, going to bed feeling stressed or angry, as well as planning or worrying when in bed [18]. Poor sleep hygiene practices among nurses were also found to have higher insomnia and anxiety issues. Understanding sleep hygiene practices among nurses working in the acute care setting is crucial to increasing awareness about the importance of sleep hygiene and developing sleep hygiene interventions to improve nurses’ sleep hygiene and sleep quality.

### Sleep Monitoring Devices

The sensitivity and accuracy of actigraphy have been determined and are commonly used in clinical settings to evaluate sleep quantity and quality [20]. It is a small wristwatch-like device that is convenient for monitoring movements when nurses are at work. Actigraphy-based personal sleep monitoring devices help users establish an objective knowledge about their sleep patterns, including sleep quality, rhythms, and duration. Although actigraphy has been identified as the standard assessment for mobile sleep monitoring, recent rapid development of personal consumer devices has highlighted the possible validity of feasible alternative approaches to actigraphy in the measurement of sleep [21]. One of the advantages of using personal consumer devices is the lower cost, with accompanying additional features, when compared to traditional actigraphy devices. Other favorable features of personal consumer sleep devices include wireless or Bluetooth connections enabling close to real-time data processing and also contain other sensor measurements, including heart rate and respiratory rate, which may provide additional data for sleep algorithms.

Previous studies have evaluated the accuracy of Apple Watch, one of the most widely used consumer-grade sleep tracking devices [22,23]. Roomkham et al [23] compared the sleep parameters recorded on Apple Watch with Philips Actiwatch Spectrum Pro. They found Apple Watch to have overall accuracy (97%) and sensitivity (99%) in detecting actigraphy-defined sleep, and adequate specificity (79%) in detecting actigraphy-defined wakefulness. Apple Watch, on average, only overestimated total sleep time by 6.31 minutes and underestimated wake after sleep onset by 5.74 minutes [23]. Although previous findings have demonstrated that Apple Watch can provide reasonably accurate estimates of total sleep time in general adult populations, evidence remains limited regarding its performance in health care workers, particularly nurses who experience irregular schedules and fragmented sleep. Most validation studies have been conducted under controlled environments, and it is unclear whether consumer wearables perform similarly in occupational settings characterized by variable sleep timing and shift-work demands. Wearable-derived sleep metrics provide objective estimates of sleep timing, duration, continuity, and sleep-stage distribution; however, these data do not capture the behavioral and environmental circumstances that may influence observed sleep patterns. Assessing sleep hygiene alongside objective sleep measures is, therefore, warranted, as it allows consideration of modifiable practices such as irregular sleep schedules, caffeine consumption, presleep cognitive or emotional arousal, and bedroom conditions that may influence wearable-recorded sleep outcomes. The integration of objective sleep monitoring with sleep hygiene assessment may offer a more contextualized understanding of nurses’ sleep health and identify behavioral targets for future sleep-focused interventions.

To address these gaps, this study aimed to evaluate the feasibility of using a commercial sleep monitoring device and to obtain preliminary evidence of agreement with a validated research-grade actigraphy device among nurses working rotating shifts. Agreement was examined by comparing total sleep time, in-bed wake time, and sleep efficiency estimates from the Apple Watch and GENEActiv. Feasibility was examined through device wear compliance and the completeness of nightly data. The secondary aim was to examine how demographic characteristics, shift-work patterns, and subjective sleep hygiene behaviors relate to objectively measured sleep parameters among nurses working rotating shifts.

## Methods

### Design

A feasibility observational cohort study design was adopted.

### Sample and Setting

A convenience sampling approach was used to recruit full-time registered nurses who are working in an acute care tertiary institution in Singapore from May 2025 to December 2025. The exclusion criteria included a history of psychiatric or neurological conditions, part-time employment, or long-term leave from work during the study period.

Sample size was not determined by a formal power analysis because this study was designed as a feasibility study [24]. For the first phase of the study, 5 participants were recruited. The focus of this phase was to conduct a local validation of Apple Watch against actigraphy, rather than to generate statistically powered estimates. Prior findings have demonstrated the relative accuracy of Apple Watch in estimating sleep and wake parameters when compared with polysomnography and actigraphy, showing moderate-to-high agreement for total sleep time and sleep efficiency [25]. In the second phase of the study, another 50 participants were recruited. This sample size was consistent with methodological recommendations for feasibility studies that emphasize obtaining preliminary estimates of outcome variability and adherence rates rather than hypothesis testing [26]. A sample of 55 participants would provide sufficient information on recruitment, attrition, and data completeness to inform the design of a future larger-scale study, while ensuring diversity of participants across demographics and shift patterns within the studied nursing population. The sample was, therefore, considered appropriate for the feasibility objectives of this study, including recruitment, device wear compliance, data completeness, and preliminary estimation of outcome variability, but was not intended to support definitive adjusted comparisons or multivariable regression modeling.

### Data Collection

#### Recruitment and Baseline Data Collection

A study invitation email was sent to all nurses at the institution to solicit their participation. The email included the study details and eligibility criteria. Weekly email reminders were sent to encourage nurses to take part in the study. Written informed consent was obtained from interested participants who approached the study team.

Upon obtaining consent, baseline data, including sociodemographic information (age, sex, race, BMI, marital status, number and ages of children, caregiving for an older family member, educational qualifications, and smoking status), were collected. Work-related information was also obtained, including shift patterns (single-shift duties included day, night, or office hours; rotating-shift duties included 2- or 3-rotation schedules comprising day, evening, and night shifts), clinical areas of work, years of experience in the current work location, job grade, and total length of service. Feasibility was assessed through device wear compliance and the extent of missing sleep data.

#### Measurement of Sleep Time and Other Vital Signs

For the first phase of the study, 5 nurses wore an Apple Watch Series 10 and a GENEActiv actigraph device (ActivInsights Ltd) during sleep for 2 weeks. All Apple Watch devices, paired iPhones (Apple), and GENEActiv devices used in this study were department-owned equipment designated for research purposes only. The Apple Watch and GENEActiv devices were provided to participants only for the duration of the monitoring period and were returned to the study team after data collection. The Apple Watch Series 10 was selected as the consumer wearable device for this feasibility study because Apple Watch is widely used, is familiar to many users, and is supported by prior validation evidence for estimating sleep duration [23]. Its wrist-worn format, automated sleep tracking, and integration of movement and physiological sensor data also made it a practical option for short-term sleep monitoring among nurses working irregular schedules. Participants were briefed by the study team to manually activate “Sleep Mode” on the Apple Watch before going to sleep and to deactivate it upon waking. For GENEActiv, participants were not required to activate or deactivate the device; sleep start and end times were derived using participants’ reported sleep and wake times to define the sleep period for analysis. Participants were reminded during the study briefing to record their sleep and wake times accurately throughout the monitoring period, and daily reminders were provided by the study team to prompt participants to document their sleep and wake times. GENEActiv has been validated against polysomnography and demonstrated reliable performance in detecting sleep and wake patterns (intraclass correlation coefficients [ICCs] of 0.79-0.85) for sleep parameters [27]. Total sleep time, in-bed wake time, and sleep efficiency were compared between GENEActiv and Apple Watch. Apple Watch data were synchronized to the paired iPhone and retrieved from the Apple Health dashboard after each participant completed the monitoring period [28]. GENEActiv raw accelerometer data were extracted in .bin format, with Actiwatch Data files or comma-separated values files generated using ActivInsights software where required [29]. The sleep report was generated using the Actigraphy sleep toolkit [30]. Sleep data were extracted from the Actigraphy sleep report after each participant completed monitoring. All extracted sleep data were reviewed by the principal investigator for completeness and consistency before analysis, while the principal investigator remained blinded to participants’ shift roster.

In the second phase of the study, additional sleep metrics from the Apple Watch were collected, including time in bed, which was measured from sleep start to sleep end; in-bed wake time; and amounts of rapid eye movement (REM), light (core), and deep sleep during the time spent in bed. Sleep efficiency was manually calculated as the percentage of time spent asleep while in bed, defined as the total sleep time divided by the time in bed and multiplied by 100.

#### Sleep Hygiene

The Sleep Hygiene Index was used to evaluate the sleep hygiene behavior of the participants. It was administered at baseline through an online survey before the start of sleep monitoring. The Sleep Hygiene Index is a self-administered 13 items designed to assess environmental and behavioral variables that could disrupt sleep prior to bedtime, including inconsistent bedtimes, feelings of stress, anger, or worry, engaging in stimulating activities in bed such as watching television, consuming caffeine, or exercising right before bedtime, as well as the comfort level of the bedroom environment [31]. Participants were asked to report on the frequency of engagement in the specific behavior (0=“never,” 1=“rarely,” 2=“sometimes,” 3=“frequently,” and 4=“always”). Total scores ranged from 0 to 52, with a higher score representing more maladaptive sleep hygiene practices. The Sleep Hygiene Index scores were presented as percentile rank, with scores above 50 indicating more disruptive sleep behaviors than average, to the extent that they would be more likely to result in poor sleep quality [31]. Scores 35 and above are considered poor sleep hygiene, 27 to 34 are considered normal, and below 26 are considered good sleep hygiene status. The Sleep Hygiene Index reported adequate reliability and validity [31]. The items from the Sleep Hygiene Index were developed based on the diagnostic criteria for insufficient sleep hygiene according to the International Classification of Sleep Disorders by the American Sleep Disorders Association [4]. The Sleep Hygiene Index reported satisfactory Cronbach α (0.66) and good test-retest reliability (*r*=0.71) [31].

### Ethical Considerations

Ethical approval was obtained from the SingHealth Centralised Institutional Review Board, Singapore (ECOS Ref: 2024‐3932). A participant information sheet detailing the study purpose, procedures, and voluntary nature was provided. Written informed consent was obtained from each participant before participation. Participants’ privacy was protected, and the confidentiality of their identities and study data were maintained throughout the study. Participants received reimbursement for the time spent participating in the study.

### Data Analysis

Data were entered and analyzed using SPSS version 25.0 (SPSS Inc).

The normality of continuous variables was examined using Shapiro-Wilk tests. Descriptive statistics were computed to summarize participants’ demographic, occupational, and behavioral characteristics. Continuous variables were reported as means with SDs, while categorical variables were summarized as frequencies and percentages.

In this feasibility study, preliminary agreement between Apple Watch and GENEActiv was assessed using the ICC for total sleep time, in-bed wake time, and sleep efficiency. Given the small validation sample (n=5), these analyses were intended to provide preliminary evidence of agreement rather than to establish measurement accuracy. ICC values below 0.50 were considered poor, 0.50 to 0.74 moderate, 0.75 to 0.90 good, and above 0.90 excellent [32]. Total sleep time, in-bed wake time, and sleep efficiency were compared directly between devices without epoch alignment [33].

Feasibility was determined by calculating device wear compliance and the completeness of nightly sleep data over the 14-night monitoring period, with higher percentages indicating better acceptability and integration of the device into participants’ routines. Wear compliance was expressed as the percentage of nights in which the device was worn. Completeness of nightly data was calculated as the percentage of nights that yielded valid sleep recordings out of the total number of nights. A valid sleep recording was defined as a night in which the device was worn during the intended sleep period and produced an interpretable sleep output with corresponding sleep start and end times. For phase 1 device-comparison analyses, a night was considered valid only when both Apple Watch and GENEActiv had usable data for the same reported sleep episode. Nights with partial recordings, implausible or incomplete sleep or wake entries, nonwear, incorrect wear, synchronization failure, or no recorded sleep output were treated as invalid and excluded from the analysis. Missing data were categorized as nonwear, incomplete or invalid sleep or wake entries, device synchronization failure, or no recorded output despite device wear.

Associations between demographic, occupational, and behavioral variables (age, sex, BMI, shift duties, work unit, parental status, total length of service, and Sleep Hygiene Index) and sleep outcomes were examined using multiple linear regression. Separate multiple linear regression analyses were performed for each sleep parameter: total sleep time, REM sleep, core and deep sleep, time in bed, in-bed wake time, and sleep efficiency. Given the feasibility design and modest sample size, all adjusted analyses were treated as exploratory. Covariates were limited to a small set of demographic, occupational, and behavioral factors considered clinically relevant to nurses’ sleep. This variable-selection strategy reduced, but did not eliminate, the risk of overfitting, particularly for categorical predictors that increased the number of model parameters. No formal adjustment was made for multiple comparisons. Accordingly, results were interpreted with reference to effect estimates, CIs, and patterns of consistency across related outcomes rather than statistical significance in isolation. Sleep-stage variables were also considered descriptively, as they represent interdependent components of total sleep time. Statistical significance was set at *P*<.05.

## Results

### Participant Characteristics

Fifty-five nurses participated, of whom 36 (65.5%) worked rotating shifts and 19 (34.5%) were on single-shift duties (Table 1). The mean age was 36 (SD 9) years, and most participants were female individuals (92.7%). The mean BMI was 24 (SD 5.3) kg/m², with 43.6% (n=24) overweight or obese (BMI>23 kg/m^2^). Around half (n=26, 47.3%) were married, and fewer had children (n=19, 34.5%). Most respondents were graduates of a baccalaureate nursing degree with honors program (60.0%). The mean total length of service was 12.2 (SD 10) years, significantly longer among nonshift nurses compared with shift nurses (15 vs 5.5 y; *P*=.02). The mean Sleep Hygiene Index score was significantly higher among shift nurses (19.8 vs 15.6; *P*=.01), indicating poorer sleep hygiene in this group. Other sociodemographic variables did not differ significantly between groups (Table 1).

**Table 1.** Demographic and occupational characteristics of nurses with different shifts.

Characteristics	All nurses (N=55)	Single-shift duty (n=19)	Rotating-shift duties (n=36)	Statistics	*P* value^a^
Age (y), mean (SD)	36 (9.0)	38 (34‐45)	35 (28‐38)	1.96 (53)^b^	.06
Sex, n (%)				—^c^	.29^d^
Male	4 (7.3)	0 (0)	6 (100.0)		
Female	51 (92.7)	19 (37.3)	32 (62.7)		
Race, n (%)				3.11 (1)^e^	.38
Chinese	40 (72.7)	16 (40.0)	24 (60.0)		
Malay	6 (10.9)	2 (33.3)	4 (66.7)		
Others (Indian, Filipino, and Eurasian)	9 (16.4)	1 (11.1)	8 (88.9)		
BMI				0.21 (53)^b^	.84
Mean (SD), kg/m^2^	24.0 (5.3)	24.2 (5.5)	23.8 (5.2)		
Normal weight (BMI<23 kg/m^2^), n (%)	31 (56.4)	9 (29.0)	22 (71.0)		
Overweight (23<BMI<27.4 kg/m^2^), n (%)	8 (14.5)	4 (50.0)	4 (50.0)		
Obese (BMI ≥27.5 kg/m^2^), n (%)	16 (29.1)	6 (37.5)	10 (62.5)		
Smoking status, n (%)				—^c^	>.99^d^
Yes	1 (1.8)	0 (0.0)	1 (100.0)		
No	54 (98.2)	19 (35.2)	35 (64.8)		
Marital status, n (%)				3.06 (2)^e^	.22
Single	26 (47.3)	6 (23.1)	20 (76.9)		
Married	26 (47.3)	12 (46.2)	14 (53.8)		
Divorced or widowed	3 (5.4)	1 (33.3)	2 (66.7)		
Have children, n (%)				2.11 (1)^e^	.15
Yes	19 (34.5)	9 (47.4)	10 (52.6)		
No	36 (65.5)	10 (27.8)	26 (72.2)		
Caring for older family member, n (%)				2.10 (1)^e^	.16
Yes	9 (16.4)	5 (55.6)	4 (44.4)		
No	46 (83.6)	14 (30.4)	32 (69.6)		
Highest educational level, n (%)				0.86 (2)^e^	.65
Institute of education diploma, polytechnic diploma, or junior college	11 (20.0)	4 (36.4)	7 (63.6)		
Bachelor’s degree	33 (60.0)	10 (30.3)	23 (69.7)		
Master’s degree and above	11 (20.0)	5 (45.5)	6 (54.5)		
Work unit, n (%)				4.87 (3)^e^	.18
Medical ward	10 (18.2)	3 (30.0)	7 (70.0)		
Surgical ward	11 (20.0)	2 (18.2)	9 (81.8)		
Department of Emergency Medicine and Operating Theater	10 (18.2)	2 (20.0)	8 (80.0)		
Others (isolation units, administrative, specialist center, community, radiology, specialist outpatient clinic)	24 (43.6)	12 (50.0)	12 (50.0)		
Job title, n (%)				1.04 (1)^e^	.31
Staff nurse or senior staff nurse	34 (61.8)	10 (29.4)	24 (70.6)		
Assistant nurse clinician or nurse clinician	21 (38.2)	9 (42.9)	12 (57.1)		
Years of working experience in current work unit, n (%)				3.78 (5)^e^	.29
≤2	11 (20.0)	3 (27.3)	8 (72.7)		
>2-5	15 (27.3)	3 (20.0)	12 (80.0)		
>5-10	9 (16.3)	3 (33.3)	6 (66.7)		
≥10	20 (36.4)	10 (50.0)	10 (50.0)		
Total length of service (y), mean (SD)	12.2 (10.0)	16.4 (8.1)	10 (10.3)	2.36 (53)^b^	.02
Sleep Hygiene Index, mean (SD)	18.3 (5.7)	15.6 (5.1)	19.8 (5.6)	−2.68 (53)^b^	.01

a Significant at *P*<.05.

b Independent 2-sample, 2-tailed *t* test (*df*).

c Not available.

d Fisher exact test was used since the expected counts were <5 in 50% of cells.

e Chi-square test (*df*).

### Agreement and Feasibility of the Apple Watch

**Figure 1.**
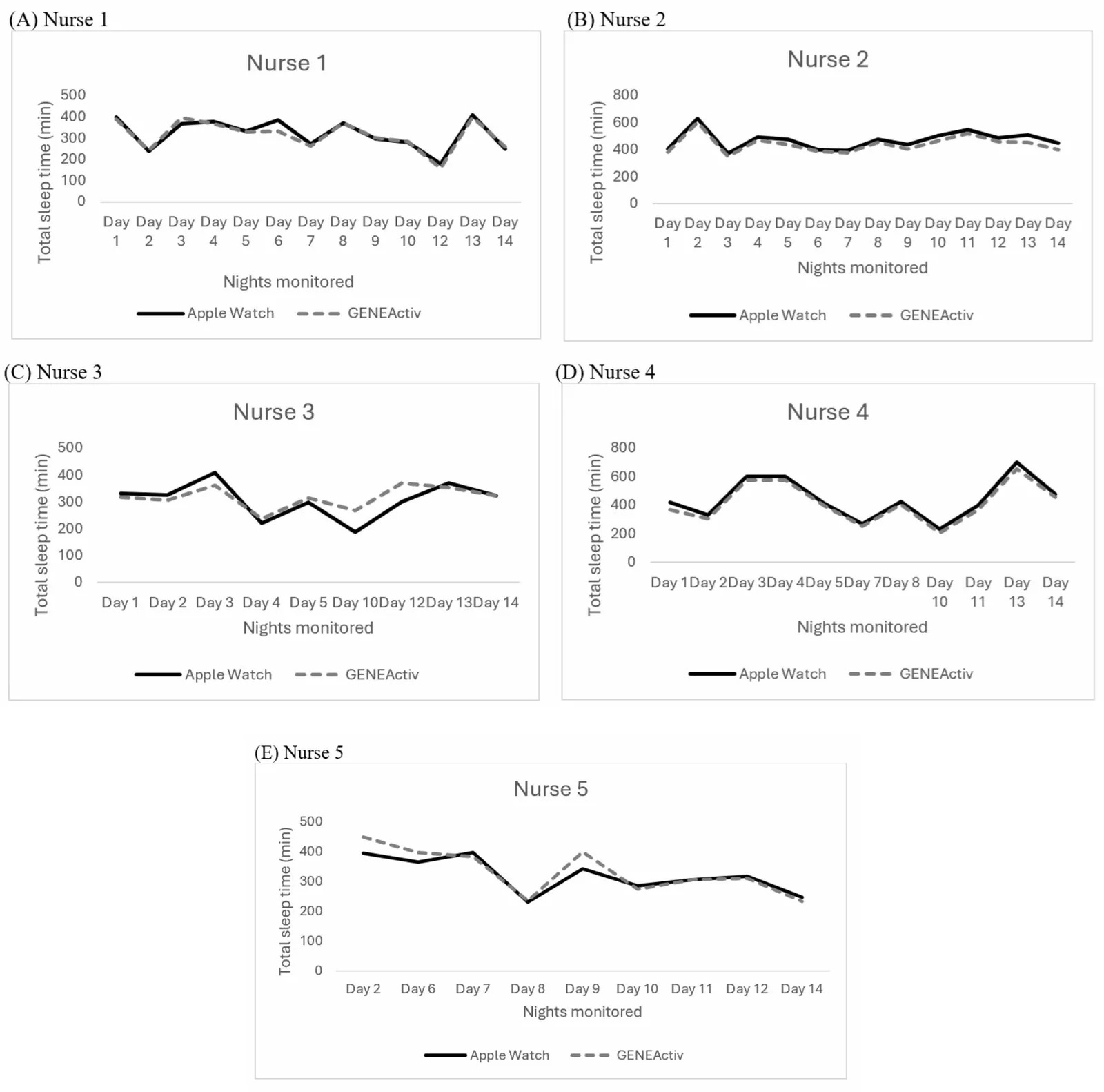
Nightly total sleep time recorded by the Apple Watch and the GENEActiv across 14 nights for (A-E) 5 nurses. Note that the missing data reflect nights when either device was not worn, was worn incorrectly, or had incomplete sleep or wake time entries, and these nights were excluded from the analysis.

Five nurses completed up to 14 consecutive nights of concurrent monitoring with the Apple Watch and GENEActiv. Apple Watch showed preliminary agreement with GENEActiv for total sleep time, in-bed wake time, and sleep efficiency (ICC=0.95, 0.72, and 0.69, respectively; Table 2). Figure 1 shows nightly total sleep time for valid paired recordings only. Across participants, the 2 devices showed closely aligned total sleep time patterns.

In phase 1, 5 nurses contributed 70 expected monitoring nights over 14 days. The Apple Watch was worn on 67 of 70 nights, giving a wear compliance of 95.7%, while 3 nights were classified as nonwear. Of the 67 nights when the Apple Watch was worn, 56 nights yielded valid Apple Watch sleep recordings, giving a data completeness rate of 83.6% among worn nights. The remaining 11 worn nights had no Apple Watch sleep output despite the device being charged and initialized before use and were therefore classified as device-related or synchronization-related data loss. For GENEActiv, 67 of 70 expected nights yielded valid recordings, while 3 nights were missing because the device was not worn. Paired device-comparison analyses were restricted to nights with valid recordings from both the Apple Watch and GENEActiv.

Across the 55 nurses, there were 770 expected nights of Apple Watch monitoring. The Apple Watch was worn on 755 nights, corresponding to 98.1% wear compliance, while 15 nights were classified as nonwear. Among the 755 worn nights, 705 yielded valid Apple Watch sleep recordings, corresponding to 93.4% data completeness among worn nights. The remaining 50 worn nights had no recorded Apple Watch sleep output despite device charging and initialization, and these nights were classified as device-related or synchronization-related data loss. Thus, missing Apple Watch data were separated into nonwear nights and nights with device-related missing output despite reported device wear.

**Table 2.** Intraclass correlation coefficients (ICC) examining preliminary agreement between the Apple Watch and GENEActiv for total sleep time, in-bed wake time, and sleep efficiency (N=5)^a^.

Sleep parameters	ICC (95% CI)	*P* value^b^
Total sleep time (min)	0.95 (0.06 to 1.00)	<.001
In-bed wake time (min)	0.72 (–0.05 to 0.97)	.05
Sleep efficiency	0.69 (–0.09 to 0.96)	.03

a ICCs represent comparisons between the Apple Watch and GENEActiv. Reliability is deemed “poor” if the ICC is <0.4, “fair” if between 0.4 and 0.59, “good” if between 0.60 and 0.74, and “excellent” if above 0.75.

b Significant at *P*<.05.

### Sleep Parameters by Shift Type

The comparison of sleep parameters between nurses working single-shift duty (day, night, or office hours) and those on rotating-shift duties (day, afternoon, and night) is reported in Table 3. Overall, the nurses obtained a mean total sleep time of 380.55 (SD 55.08) minutes. Nurses on rotating shifts recorded a mean total sleep time of 382.03 (SD 55.92) minutes compared with 377.76 (SD 55.85) minutes among single-shift nurses; this difference was not significant in the unadjusted analysis (mean difference=−4.27, 95% CI −35.87 to 27.33 min; *P*=0.79) and remained nonsignificant after adjustment for age, sex, BMI, presence of children, workplace, total length of service, and sleep hygiene score (adjusted mean difference=−7.38, 95% CI −40.74 to 25.99 min; *P*=.66). Similarly, no significant group differences were found for REM, core, or deep sleep in either unadjusted or adjusted analyses (*P*>.05).

The mean time in bed for all nurses was 401.45 (SD 54.57) minutes, the mean wake time in bed was 19.79 (SD 13.76) minutes, and the average sleep efficiency was 94.77% (SD 4.11%). Mean time in bed was similar between nurses on single shifts (402.46, SD 55.09 min) and rotating shifts (400.92, SD 55.07 min), with no statistically significant differences before or after covariate adjustment. Similarly, in-bed wake time and sleep efficiency did not differ significantly by shift type.

**Table 3.** Sleep parameters of nurses with different shifts.

Sleep parameters	All nurses (N=55), mean (SD)	One-shift duty (day, night, or office hours; n=19), mean (SD)	Rotating-shift duties (morning, afternoon, and night; n=36), mean (SD)	Unadjusted mean difference^a^ (95% CI)	*t* test (*df*)	*P* value	Adjusted mean difference^b^ (95% CI)	*P* value ^c^
Total sleep time (min)	380.55 (55.08)	377.76 (55.85)	382.03 (55.92)	−4.27 (−35.87 to 27.33)	–0.27 (53)	.79	−7.38 (−40.74 to 25.99)	.66
Rapid eye movement	80.63 (20.26)	83.89 (18.51)	78.90 (21.17)	4.99 (−6.56 to 16.54)	0.87 (53)	.39	5.58 (−6.42 to 17.58)	.35
Core (light)	240.86 (42.07)	238.20 (45.76)	242.27 (40.60)	−4.07 (−28.20 to 20.06)	–0.34 (53)	.74	−7.81 (−34.08 to 18.46)	.55
Deep	48.29 (10.02)	49.76 (8.77)	47.52 (10.66)	2.24 (−3.48 to 7.96)	0.79 (53)	.44	3.13 (−2.41 to 8.67)	.26
Time in bed (min)	401.45 (54.57)	402.46 (55.09)	400.92 (55.07)	1.54 (−29.79 to 32.86)	0.10 (53)	.92	−0.23 (−33.53 to 33.08)	.99
In-bed wake time (min)	19.79 (13.76)	23.20 (20.08)	17.99 (8.66)	5.21 (−2.56 to 12.98)	1.35 (53)	.18	5.04 (−4.18 to 14.25)	.28
Sleep efficiency (%; total sleep time/time in bed)×100	94.77 (4.11)	93.90 (5.03)	95.22 (3.53)	−1.33 (−3.66 to 1.00)	–1.14 (53)	.26	−1.78 (−4.47 to 0.90)	.19

a Independent 2-sample, 2-tailed *t* test.

b Adjusted for age, sex, BMI, presence of children, workplace, total length of service, and sleep hygiene score using multiple linear regression.

c Significant at *P*<.05.

### Associations Between Selected Variables and Sleep Outcomes

Results from multiple linear regression analyses examining exploratory associations between selected variables and sleep parameters derived from Apple Watch devices were presented in Table 4. Given the modest sample size relative to the number of predictors and outcomes, these findings should be regarded as exploratory and hypothesis-generating. After adjustment for age, sex, BMI, shift duties, parental status, work unit, total length of service, and Sleep Hygiene Index, several associations were observed. Higher BMI was associated with shorter total sleep time (unstandardized coefficient *B*=−3.76, 95% CI −6.70 to −0.81 min/kg/m²; *P*=.01), reduced REM sleep duration (unstandardized coefficient *B*=−1.18, 95% CI −2.24 to −0.12 min; *P*=.03), and shorter core (light) sleep duration (unstandardized coefficient *B*=−2.72, 95% CI −5.04 to −0.40 min; *P*=.02). BMI was also associated with reduced time in bed (unstandardized coefficient *B*=−4.05, 95% CI −6.99 to −1.11 min; *P=*.008). Age was negatively associated with deep sleep duration, with older age associated with less deep sleep (unstandardized coefficient *B*=−1.00, 95% CI −1.65 to −0.35 min/y; *P*=.003). Overall, sex, parental status, work unit, total length of service, and Sleep Hygiene Index were not significantly associated with total sleep time, REM sleep, deep sleep, in-bed wake time, or sleep efficiency (*P*>.05).

**Table 4.** Multiple linear regression analyses examining sleep parameters among nurses (N=55)^a^.

Characteristics	Total sleep time (min)		REM^b^ (min)		Core (min)		Deep (min)		Time in bed (min)		In-bed wake time (min)		Sleep efficiency (%)	
	*B* (95% CI)	*P* value^c^	*B* (95% CI)	*P* value^c^	*B* (95% CI)	*P* value^c^	*B* (95% CI)	*P* value^c^	*B* (95% CI)	*P* value^c^	*B* (95% CI)	*P* value^c^	*B* (95% CI)	*P* value^c^
Age (y)	−0.57 (−4.45 to 3.32)	.77	−0.69 (−2.09 to 0.71)	.33	0.87 (−2.20 to 3.93)	.57	−1.00 (−1.65 to 0.35)	.003	0.16 (−3.72 to 4.04)	.93	0.29 (−0.79 to 1.36)	.59	−0.15 (−0.47 to 0.16)	.33
Sex (female)	35.55 (−24.61 to 95.71)	.24	7.22 (−14.42 to 38.86)	.51	28.57 (−18.81 to 75.94)	.23	6.02 (−3.97 to 16.00)	.23	25.95 (−34.12 to 86.01)	.39	−6.43 (−23.04 to 10.19)	.44	2.95 (−0.47 to 0.16)	.33
BMI (kg/m^2^)	−3.76 (−6.70 to −0.81)	.01	−1.18 (−2.24 to −0.12)	.03	−2.72 (−5.04 to −0.40)	.02	−0.32 (−0.81 to 6.72)	.19	−4.05 (−6.99 to −1.11)	.008	−0.06 (−0.87 to 0.75)	.88	0.03 (−0.21 to 0.26)	.83
Have children (yes)	11.26 (−22.95 to 45.48)	.51	7.77 (−4.54 to 20.08)	.21	7.91 (−19.04 to 35.85)	.56	1.04 (−4.64 to 6.72)	.71	10.23 (−23.94 to 44.39)	.55	−1.47 (−10.92 to 7.98)	.76	0.38 (−2.37 to 3.13)	.78
Work unit	1.17 (−11.51 to 13.84)	.85	0.77 (−3.79 to 5.33)	.74	−2.20 (−12.18 to 7.78)	.66	0.32 (−1.78 to 2.43)	.76	1.58 (−11.07 to 14.23)	.80	0.61 (−2.99 to 4.11)	.73	−0.01 (−1.03 to 1.01)	.98
Total length of service (y)	−0.80 (−4.39 to 2.79)	.66	−0.18 (−1.47 to 1.11)	.78	−1.24 (−4.07 to 1.59)	.38	0.50 (−0.10 to 1.09)	.10	−1.20 (−4.78 to 2.39)	.51	−0.04 (−1.03 to 0.95)	.94	0.06 (−0.23 to 0.35)	.70
Sleep Hygiene Index	−1.31 (−4.13 to 1.51)	.36	−0.38 (−1.40 to 0.63)	.45	−1.15 (−3.37 to 1.07)	.30	0.03 (−0.43 to 0.50)	.88	−1.02 (−3.84 to 1.80)	.47	0.07 (−0.71 to 0.85)	.86	−0.11 (−0.33 to 0.12)	.35

a Values are unstandardized coefficients (*B*) with 95% CIs and *P* values. Each column represents a separate regression model adjusted for all listed predictors; reference categories: male (sex) and no children.

b REM: rapid eye movement.

c Significant at *P*<.05.

## Discussion

### Principal Findings

This study evaluated the feasibility of consumer wearable sleep monitoring among nurses and obtained preliminary evidence of agreement with validated actigraphy in a small validation sample for total sleep time, in-bed wake time, and sleep efficiency. These findings are consistent with previous studies showing that consumer sleep technologies can provide reasonably accurate estimates of total sleep duration compared with research-grade actigraphy devices [33,34]. Much of the existing literature on nurses’ sleep has relied on subjective measures, which capture perceived sleep quality but may not accurately quantify sleep duration, sleep-wake patterns, or awakenings. Previous studies have also shown limited agreement between subjective sleep reports and objective measures among nurses [35]. Accordingly, actigraphy and wearable devices are increasingly used to support continuous sleep assessment during clinical schedules [36], although further validation and longer monitoring periods are needed to establish their accuracy and utility in occupational settings [37].

Wear compliance and data completeness supported the feasibility of Apple Watch monitoring in this setting, although some data loss resulted from nonwear or technical issues, as expected in real-world wearable studies [38,39]. Beyond measurement, wearable feedback may complement sleep education by increasing awareness of sleep routines, consistent with behavioral self-efficacy and behavior change models [40,41].

Nurses slept an average of 381 minutes, or approximately 6.4 hours per night, which is below the recommended 7 to 9 hours for adults [42]. Although sleep efficiency was high (94.77%), this should not be interpreted as unequivocal evidence of robust sleep quality. In the context of short total sleep duration and limited time in bed, elevated sleep efficiency may instead reflect constrained sleep opportunity, whereby nurses slept for most of the limited time available rather than achieving sufficient or restorative sleep. This finding is consistent with studies reporting a high prevalence of poor sleep quality and disrupted sleep among nurses in Asian and shift-working populations [43-46]. Insufficient sleep has been associated with fatigue, impaired cognitive and psychomotor performance, burnout, emotional dysregulation, and occupational fatigue, underscoring the clinical relevance of the short sleep duration observed in this cohort [47-51].

Contrary to expectations, shift duty status was not significantly associated with any sleep parameter after covariate adjustment. Instead, BMI showed the most consistent associations, with higher BMI associated with shorter total sleep time, reduced REM and core sleep, and less time in bed. Although shift nurses reported poorer sleep hygiene than nonshift nurses, Sleep Hygiene Index scores were not independently associated with sleep outcomes in the adjusted models. These findings suggest that individual lifestyle and behavioral factors may be associated with sleep outcomes beyond work schedule alone. Similar observations have been reported in studies showing associations between daily routines, diet, sleep timing around shifts, and sleep quality among nurses [52]. Multiple correlated sleep outcomes were examined across several predictors without adjustment for multiple comparisons; therefore, the regression findings should be interpreted cautiously, with greater emphasis on effect sizes, CIs, and consistency across related outcomes rather than isolated *P* values.

Age also emerged as a significant determinant of sleep outcomes, with older nurses showing reduced deep sleep duration. This pattern is consistent with well-established evidence showing that deep sleep declines across adulthood as a result of age-related changes in cortical activity and sleep homeostasis [53]. Recent studies using both consumer wearables similarly reported a reduction in deep sleep with age among working adults, including health care staff [38,54]. Given that deep sleep contributes to physiological recovery, cognitive functioning, and metabolic regulation, reduced deep sleep among older nurses may have important consequences for fatigue, resilience, and day-to-day clinical effectiveness. Supporting older nurses through workplace policies and tailored sleep-health interventions may be an important component of broader efforts to support and maintain the long-term sustainability of the nursing workforce.

### Implications for Nursing Practice

These findings have practice-relevant implications for the nursing workforce. Organizational strategies should prioritize sleep opportunity and fatigue risk management through rostering review, protected rest periods, and minimization of excessive consecutive shifts or overtime. Older nurses may also benefit from tailored fatigue-management support.

Wearable sleep tracking may complement staff well-being initiatives by providing objective feedback alongside sleep education, particularly for nurses who underestimate sleep disruption based on self-report alone. Accordingly, any recommendation for broader Apple Watch use should be balanced against considerations of cost, scalability, and local implementation needs, including whether lower-cost wearables, validated sleep questionnaires, or targeted actigraphy may provide a more proportionate approach for workplace sleep-health initiatives.

Third, the association between higher BMI and poorer sleep parameters underscores the importance of adopting a holistic approach to health promotion within the nursing workforce. Initiatives that support physical activity, healthy nutrition, weight management, and stress regulation may contribute not only to improved sleep outcomes but also to overall well-being.

Finally, poorer sleep hygiene among shift nurses indicates the value of incorporating practical sleep-hygiene guidance into staff well-being programs across shift types.

### Limitations

Several limitations should be considered. First, the validation component involved only 5 nurses; therefore, the device-comparison findings should be interpreted as preliminary feasibility evidence rather than established measurement accuracy. Although small samples are acceptable in feasibility studies, recruiting the cohort from a single institution may limit generalizability. An a priori power analysis was not conducted because the study primarily evaluated feasibility. The adjusted analyses included several covariates and multiple interdependent sleep outcomes relative to the sample size and may therefore have been underpowered, overfitted, or imprecise. As no adjustment for multiple comparisons was applied, statistically significant findings should be regarded as exploratory and interpreted cautiously until confirmed in adequately powered studies with prespecified analytic plans.

Self-selection bias may also have occurred, as nurses who volunteered may have had a greater interest in sleep monitoring or have perceived sleep difficulties. Self-reported questionnaires, including the Sleep Hygiene Index, may be affected by recall or social desirability bias. The nonsignificant associations between Sleep Hygiene Index scores and objective sleep outcomes should be interpreted cautiously, as they may reflect measurement limitations, restricted variability in this relatively homogeneous cohort, and limited power to detect small associations. In addition, manual inspection of sleep data was performed only by the principal investigator; although the principal investigator was blinded to participants’ shift rosters, the absence of independent interrater verification may have introduced subjective judgment in determining data completeness and consistency.

Chronotype was not assessed in this study, although individual circadian preference may influence tolerance for shift work and could partly explain why shift status was not independently associated with sleep outcomes. Future studies should examine whether chronotype moderates the relationship between shift schedules and sleep outcomes to guide chronobiologically informed scheduling. Finally, although total sleep time, in-bed wake time, and sleep efficiency were compared between the Apple Watch and the GENEActiv, device-specific sleep-window definitions, algorithms, scoring rules, and firmware updates may affect wake-related estimates and reproducibility over time.

Despite these limitations, this study provides objective sleep data among nurses in an Asian context, as well as preliminary evidence supporting the feasibility of Apple Watch–based monitoring and its agreement with validated actigraphy for selected sleep parameters. The observed associations remain exploratory given the observational design and modest sample size.

### Conclusions

This study showed that nurses obtained less sleep than recommended, underscoring concerns about sleep opportunity and recovery in this workforce. The Apple Watch provided preliminary evidence of feasibility and agreement with GENEActiv for total sleep time, in-bed wake time, and sleep efficiency in a small validation sample. BMI and age were associated with sleep outcomes in exploratory analyses. These findings support workplace strategies that combine sleep education, sleep hygiene training, and broader fatigue-management efforts to promote healthier sleep among nurses.

## Abbreviations

ICC: intraclass correlation coefficient
REM: rapid eye movement
